# Associations of night sleep duration and daytime napping with diabetic retinopathy in patients with type 2 diabetes

**DOI:** 10.3389/fendo.2025.1565508

**Published:** 2025-06-24

**Authors:** Lei Xi, Xiaohui Sun, Zhimin Feng, Yanan Cao

**Affiliations:** Department of Endocrine and Metabolic Diseases, Shanghai Institute of Endocrine and Metabolic Diseases, National Clinical Research Center for Metabolic Diseases (Shanghai), Ruijin Yangtze River Delta Health Institute, Research Unit of Clinical and Basic Research on Metabolic Diseases of Chinese Academy of Medical Sciences, Wuxi Branch of Ruijin Hospital, Ruijin Hospital, Shanghai Jiao Tong University School of Medicine, Shanghai, China

**Keywords:** sleep, napping, type 2 diabetes, diabetic retinopathy, metabolic factors

## Abstract

**Aims:**

The purpose of this study was to evaluate the relationship between night sleep duration, daytime napping, and diabetic retinopathy (DR) in type 2 diabetes (T2D) patients and to explore the potential mediating role of metabolic factors.

**Methods:**

In this cross-sectional, retrospective study, night sleep and napping were assessed according to the standardized questionnaire. The metabolic factors in the examination were systolic blood pressure (SBP), diastolic blood pressure (DBP), body mass index (BMI), and HbA1c. Multivariate logistic regression and stratified and conjoint analysis were carried out. In addition, causal mediation analysis was performed to explore the mediating role.

**Results:**

A total of 2,433 patients [mean (SD) age, 55.82 (11.66) years; 40.07% women] were included in the final analysis. The prevalence of DR was 15.95%. Compared with reference groups, patients with long sleep [odds ratio (OR), 1.31, 95% confidence interval (CI), 1.01–1.70] and long nap (1.09, 1.04–1.23) were both associated with DR, and stratified analysis showed that this association varied among different sex and diabetes duration groups. Conjoint analysis showed that patients with both long sleep and long naps had a significantly increased risk of DR (1.75, 1.13–2.71). Mediation analysis showed that metabolic factors partially mediated this association between night sleep, naps, and DR, contributing to 9.8% and 16.3% of the total effects, respectively.

**Conclusions:**

Long sleep and long nap were associated with DR, and male patients with T2D with a shorter course (<5 years) especially need to be vigilant. The effects of night sleep and naps on DR could be superimposed, and metabolic factors partially explain the underlying mechanism.

## Introduction

Diabetes, one of the most common chronic diseases, is gaining more and more attention. The International Diabetes Federation (IDF) reported that 8.3% of adults, approximately 382 million, worldwide have diabetes, and its incidence continues to increase. It is expected that in 2030, the number of patients with diabetes will exceed 500 million globally, among which the most common type is type 2 diabetes (T2D), accounting for 85.0%–95.0% (1–3). Among complications, diabetic retinopathy (DR), the leading cause of preventable blindness in adults, affects approximately 40% of all patients, seriously reducing their quality of daily life and bringing heavy burden to their families and society (4, 5).

Previous studies have shown that anomalous sleep duration is associated with higher risk of T2D (6, 7). However, limited studies investigated the relationship between sleep phenotypes, including night sleep and daytime napping with DR, and results were not consistent. A cross-sectional survey showed that long sleep independently associated with DR (8); a Korean study has highlighted gender differences (9), while other studies did not find a correlation (10, 11). Critically, prior research often examined night sleep and daytime napping in isolation, neglecting their potential additive or synergistic effects on DR risk. This gap is noteworthy because disrupted circadian rhythms from prolonged nighttime sleep abnormalities may drive compensatory daytime napping, collectively exacerbating metabolic dysregulation (e.g., insulin resistance and sympathetic activation) that accelerates microvascular damage in DR (12–14). Furthermore, daytime napping could reflect poor sleep quality at night or fragmented sleep architecture, which may independently contribute to retinal hypoxia and inflammation (15). In addition, the mechanisms underlying sleep-associated DR are not fully understood, and modifiable behavioral factors, including metabolic factors, physical activity, and diet, are the possible risk factors (16–18). Among them, blood pressure, body mass index (BMI), and HbA1c have been found to be closely related to sleep and DR (19, 20).

Based on these observations, our study aimed to investigate the potential relationship between sleep phenotypes, including night sleep duration and daytime napping, and DR among Chinese patients with T2D. In addition, metabolic factors (blood pressure, BMI, and HbA1c) were also included in the analysis to identify potential mediators.

## Materials and methods

### Study population and design

The study population is composed of the 2,634 patients with T2D who were assessed at the National Metabolic Management Center (MMC) in Ruijin Hospital (Shanghai, China) between June 2017 and June 2021. As described in our previous research (21), MMC has built standard services both inside and out of the hospital and patients can enjoy one-stop care to receive a comprehensive series of services from registration, tests, evaluation, prescriptions, to health education. The study was approved by the Ethics Committee of Ruijin Hospital and obtained written informed consent from each participant.

The inclusion criteria were as follows: (1) adults diagnosed with T2D aged 18 years and older and (2) complete clinical data. The exclusion criteria were as follows: (1) lack of sleep data and (2) lack of evaluation and documentation of DR.

### Data collection and covariates

Data collection was performed by MMC medical staff according to a standard protocol. Detailed information on demographic characteristics, medical record, and lifestyle behaviors was obtained. Specifically, age, sex, systolic blood pressure (SBP), diastolic blood pressure (DBP), BMI, and duration of diabetes were gathered based on the MMC system. Glycated hemoglobin (HbA1c) levels were performed in the central clinical laboratory of Ruijin Hospital by automated analyzers (22, 23). Smoking was defined as current smoking or quitting smoking for not more than 12 months and nondrinker was defined as a person who never drank alcohol or quit. The covariates included in the regression model include the above indicators, which are based on theoretical evidence of previous studies (8–11, 18–20) and statistical significance.

### Assessment of night sleeping duration, napping, and DR

Habitual nocturnal sleep duration and daytime napping duration were obtained by trained medical staff according to a standardized process of MMC through a validated questionnaire at the initial visit. The specific questions regarding sleep phenotypes are as follows: (1) “How many hours do you take a daytime napping every day at noon?“; (2) “How many hours do you sleep every night (Record the time of falling asleep and waking up)?”. Time recording adopts a 24-h system, and the answer to this question is accurate to the minute and all results of sleep duration have been converted into hours (e.g., 8 h 30 min = 8.5 h). Based on the night sleep duration, we divided the patients into three groups: short (<7 h), middle (7–8 h), and long (>8 h) sleep. Based on the napping duration, we also divided the patients into three groups: no nap (0 h), short nap (>0–<1 h), and long nap (≥1 h). According to the previous studies, middle sleep and no nap were used as references (24, 25).

Combined with fundus examination and the international DR grading standard in 2003 (22, 26), DR was defined as moderate non-proliferative diabetic retinopathy (NPDR) or worse, or clinical events including vitreous/preretinal hemorrhage, clinically significant macular edema, treatment with laser or anti-vascular endothelial growth factor therapy, vitrectomy, and diabetes-related blindness and controls were defined as without DR, or with mild NPDR. DR was confirmed or excluded with combined diagnostic tools as direct fundus examination under slit lamp, fundus photography, optical coherence tomography (Triton DRI-OCT, Topcon, Inc., Tokyo, Japan), and fundus fluorescence angiography (FFA) or OCT angiography (OCTA). At least one standard non-dilated pupil, macula-centered color, and non-stereoscopic retinal fundus image with 45° visual field was obtained from each participant per eye. The integrated results were evaluated by experienced and trained ophthalmologists and finally reviewed by fundus experts.

### Statistical analysis

All statistical analyses were conducted using R software (version 4.1.2) and IBM SPSS Statistics 24.0 (SPSS Inc., Chicago, IL, United States). Continuous variables were expressed as mean with SD, and categorical variables were presented as numbers (proportions). General characteristics of the participants were shown by groups of night sleep duration. For continuous variables, ANOVA was conducted, and *p*-values were used to assess group differences. For categorical variables, chi-square tests were employed. For variables with missing values (≤3% missingness), median imputation was applied to continuous variables, while mode imputation was used for categorical variables. Multivariate logistic regression analyses were applied to examine the associations of night sleep duration, daytime napping, and DR. Odds ratios (ORs) were adjusted for age, sex, smoking, drinking, SBP, DBP, BMI, HbA1c, and duration of diabetes. The R package “mediation” was used for mediation analysis. Mediation effects were analyzed using the mediation package (v4.5.0) in R 4.1.2. The quasi-Bayesian Monte Carlo method with 5,000 simulations was adopted to obtain the estimates for the average causal mediation effect, average direct effect, and total effect of potential mediators. In bilateral tests, *p* < 0.05 indicated statistically significance.

## Results

### Patient characteristics

After excluding missing values for night sleep duration and records of DR, a total of 2,433 patients were included in this study and general characteristics are shown in **Table 1**. Among all participants, there were 1,458 men (59.93%) and 975 women (40.07%). Average age of visit and duration of diabetes were 55.82 and 8.05 years, respectively. Of the total participants, 89.79% were nondrinkers and a total of 15.95% of patients presented with DR; 21.17%, 51.99%, and 26.84% reported night sleeping for <7, 7–8, and >8 h, respectively. The long sleep group was more likely to be nondrinkers (90.78%), have a longer course of diabetes (8.57 years), and have a higher proportion of DR (19.30%), compared with the middle and short sleep group. In addition, there were no significant differences in age of visit, smoking, blood pressure, and HbA1c among the three groups of patients.

**Table 1:** Characteristics of the participants at baseline.

Variables	Total	Short sleep	Middle sleep	Long sleep	*p*-value
		<7 h/night	7–8 h/night	>8 h/night	
*N*	2,433	515	1,265	653	
Age, years	55.82 ± 11.66	55.83 ± 11.47	55.40 ± 11.65	56.64 ± 11.79	0.088
Women, *n* (%)	975 (40.07)	200 (38.83)	499 (39.45)	276 (42.27)	0.398
Ideal smoking status*, *n* (%)	1,827 (75.68)	386 (75.54)	955 (76.22)	486 (74.77)	0.907
Nondrinker^#^, *n* (%)	2,172 (89.79)	445 (86.74)	1,136 (90.52)	591 (90.78)	0.004
Duration of diabetes, years	8.05 ± 7.51	8.33 ± 7.69	7.66 ± 7.15	8.57 ± 8.01	0.027
Systolic blood pressure, mmHg	129.61 ± 17.48	130.53 ± 18.56	129.26 ± 17.04	129.56 ± 17.45	0.380
Diastolic blood pressure, mmHg	74.76 ± 10.81	75.34 ± 10.95	74.58 ± 10.73	74.65 ± 10.84	0.391
HbA1c, %	7.82 ± 1.66	7.83 ± 1.64	7.78 ± 1.69	7.90 ± 1.60	0.375
Body mass index, kg/m^2^	25.79 ± 3.76	26.21 ± 3.98	25.73 ± 3.69	25.58 ± 3.71	0.013
Night sleep duration, h	7.58 ± 1.24	5.85 ± 0.78	7.53 ± 0.44	9.03 ± 0.67	<0.001
Daytime napping, h/day	0.46 ± 0.59	0.47 ± 0.59	0.45 ± 0.58	0.46 ± 0.62	0.940
None	1,223 (50.27)	242 (47.00)	634 (50.12)	347 (53.14)	
Short nap	518 (21.29)	126 (24.46)	272 (21.50)	120 (18.38)	
Long nap	692 (28.44)	147 (28.54)	359 (28.38)	186 (28.48)	
Diabetic retinopathy, *n* (%)	388 (15.95)	78 (15.15)	184 (14.55)	126 (19.30)	0.023

Data are mean ± SD or *n* (%). *Ideal smoking status was defined as not smoking or quitting smoking for more than 12 months. ^#^Nondrinker was defined as a person who never drank alcohol or quit. Short nap was defined as duration of daytime napping less than 1 h. Long nap was defined as duration of daytime napping no less than 1 h.

### Associations of sleep phenotypes with DR events

**Table 2** shows the multivariate logistic regression analysis with DR as dependent variable in patients with T2D. According to the sleep groups, compared with those with middle sleep, patients with long sleep had a higher risk of DR (OR 1.39, 95% CI 1.08–1.79) when adjusted for age and sex (model 1). Moreover, such a relationship was still established when further adjusted for smoking, alcohol consumption, SBP, DBP, BMI, HbA1c, and duration of diabetes (1.31, 1.01–1.70, model 2). In comparison, there was no correlation between short sleep and DR (1.02, 0.75–1.38, *p* = 0.913). As for daytime napping, long nap was associated with the risk of DR (model 1, 1.14, 1.08–1.28; model 2, 1.09, 1.04–1.23) after adjusting variables, and there was no correlation between short nap and DR (0.98, 0.72–1.32, *p* = 0.870), compared with the reference group.

**Table 2:** Associations of sleep phenotypes with DR events in patients with type 2 diabetes.

Variables	Model 1	*p*-value	Model 2	*p*-value
Night sleep duration				
Middle sleep	1.00 (Ref)			
Short sleep	1.07 (0.80–1.43)	0.664	1.02 (0.75–1.38)	0.913
Long sleep	1.39 (1.08–1.79)	0.011*	1.31 (1.01–1.70)	0.047*
Daytime napping				
No nap	1.00 (Ref)			
Short nap	1.02 (0.76–1.37)	0.900	0.98 (0.72–1.32)	0.870
Long nap	1.14 (1.08–1.28)	0.012*	1.09 (1.04–1.23)	0.023*

Values are expressed as odds ratio (95% confidence interval). Model 1 was adjusted for age and sex; model 2 was further adjusted for smoking, alcohol consumption, SBP, DBP, BMI, HbA1c, and duration of diabetes. Middle sleep and no nap were used as references, respectively. *Significant at *p* < 0.05.

**Table 3** shows stratified analysis of the association of long sleep and long nap with DR. When the independent variable was night sleep, diabetes duration <5 years and male groups were positively associated with the risk of DR (1.62, 1.04–2.52; 1.53, 1.09–2.16), while there was no statistical significance between the groups of age and HbA1c after adjusting for age, sex, smoking, alcohol consumption, SBP, DBP, BMI, HbA1c, and duration of diabetes. When the independent variable was long nap, only diabetes duration <5 years was positively associated with the risk of DR (1.26, 1.03–1.40). In addition, the results of the conjoint analysis showed that patients with long sleep and long nap were positively associated with DR (1.75, 1.13–2.71, **Table 4**) compared with patients with no nap and middle sleep.

**Table 3:** Stratified analysis of the association of long sleep and long nap with DR.

Variables	Long sleep			Long nap		
	OR (95% CI)	*p*-value	*p* for interaction	OR (95% CI)	*p*-value	*p* for interaction
Sex			0.908			0.423
Male	1.53 (1.09–2.16)	0.014		0.99 (0.70–1.39)	0.929	
Female	1.00 (0.66–1.52)	0.994		1.13 (0.74–1.74)	0.571	
Age			0.151			0.820
<60 year	1.28 (0.87–1.87)	0.208		0.96 (0.64–1.44)	0.829	
≥60 year	1.36 (0.94–1.96)	0.104		1.16 (0.80–1.68)	0.442	
Diabetes duration			<0.001			<0.001
<5 years	1.62 (1.04–2.52)	0.032		1.26 (1.03–1.40)	0.022	
≥5 years	1.23 (0.89–1.70)	0.211		1.18 (0.85–1.64)	0.318	
HbA1c			0.027			0.232
<7%	1.27 (0.79–2.05)	0.325		1.04 (0.62–1.74)	0.885	
≥7%	1.25 (0.91–1.72)	0.170		1.11 (0.80–1.53)	0.535	

Values are expressed as odds ratio (95% confidence interval). Model was adjusted for age, sex, smoking, alcohol consumption, SBP, DBP, BMI, HbA1c, and duration of diabetes. Middle sleep and no nap were used as references in different subgroups, respectively.

**Table 4:** Conjoint analysis of night sleep duration and daytime napping on DR.

Variables	Night sleep duration		
	Middle sleep	Short sleep	Long sleep
Daytime napping			
No nap	1.00 (Ref)	0.96 (0.61–1.50)	1.28 (0.89–1.86)
Short nap	1.04 (0.68–1.60)	1.16 (0.67–2.01)	0.95 (0.53–1.69)
Long nap	0.99 (0.67–1.45)	0.99 (0.58–1.69)	1.75 (1.13–2.71)*

Values are expressed as odds ratio (95% confidence interval). Model was adjusted for age, sex, smoking, alcohol consumption, SBP, DBP, BMI, HbA1c, and duration of diabetes. Group of middle sleep and no nap was used as reference.*Significant at *p* < 0.05.

### Mediation effects of metabolic factors on night sleep, napping–DR risk

**Figure 1:**
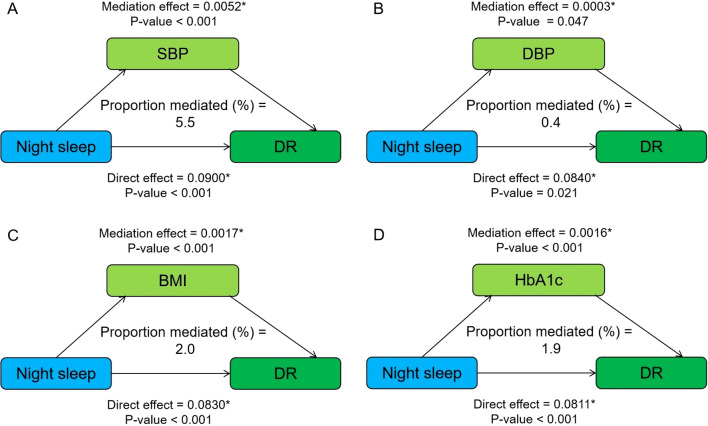
Mediation analysis of metabolic factors, including SBP **(A)**, DBP **(B)**, BMI **(C)**, and HbA1c **(D)** on the relationship between night sleep duration and DR. Model was adjusted for age, sex, smoking, alcohol consumption, SBP, DBP, BMI, HbA1c, and duration of diabetes. When a metabolic factor itself acts as a mediator in turn, excluding itself as a covariate. * Significant at p < 0.05.

**Figure 2:**
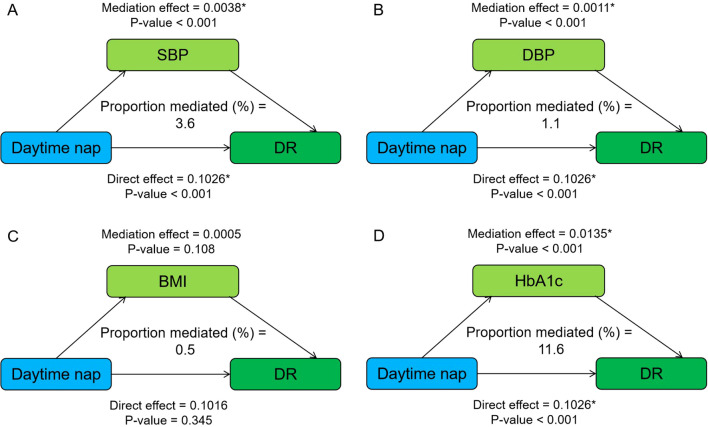
Mediation analysis of metabolic factors, including SBP **(A)**, DBP **(B)**, BMI **(C)**, and HbA1c **(D)** on the relationship between daytime napping and DR. Model was adjusted for age, sex, smoking, alcohol consumption, SBP, DBP, BMI, HbA1c, and duration of diabetes. When a metabolic factor itself acts as a mediator in turn, excluding itself as a covariate. * Significant at p < 0.05.

**Figure 1** shows the mediating role of metabolic factors in the relationship between night sleep duration and DR. Model was adjusted for age, sex, smoking, alcohol consumption, SBP, DBP, BMI, HbA1c, and duration of diabetes. When a metabolic factor itself acts as a mediator in turn, excluding itself as a covariate. All four metabolic factors significantly mediated the association between long sleep and DR, with SBP, DBP, BMI, and HbA1c explaining 5.5%, 0.4%, 2.0%, and 1.9% of the association, respectively (both *p* < 0.05). In addition, the relationship between daytime napping and DR was partially mediated by SBP, DBP, and HbA1c, accounting for 3.6%, 1.1%, and 11.6%, respectively, while the mediating role of BMI tended to be non-significant (**Figure 2**).

## Discussion

In this study, associations between night sleep, daytime napping, and DR in patients with T2D were investigated. Our study indicated that long sleep and long nap were associated with DR, and this association was more significant in men and in the diabetes duration < 5 years group. The results of the joint analysis showed that the effects of night sleep and nap on DR could be superimposed, and that patients with long sleep and long naps were positively associated with DR. In addition, metabolic factors, including SBP, DBP, BMI, and HbA1c, partially explain the mechanism between night sleep, daytime napping, and DR.

In the present study, 15.95% of patients suffered from DR, and among them, 40.07% were female, which was similar to previous studies (27–29). In recent years, a growing number of studies have confirmed that sleep was closely related to DR (12, 30–33). The vast majority of research focused on the impact of night sleep duration and sleep quality on DR, with substantial heterogeneity observed across studies. Long sleep was associated with the risk of DR, while short sleep was controversial. There are many sources of heterogeneity, and differences in race, age, BMI, and sleep assessment methods may partly explain it. A meta-analysis involving six studies showed that association between short sleep and DR was significant in those ≥60 years, while there was no significance in the BMI subgroup analysis (12). It should be acknowledged that the sample size of short sleep patients in previous studies and our research is relatively small, and further research is needed to confirm our results. In our study, long sleep duration was positively associated with DR, and this association was more significant in men and in the diabetes duration < 5 years group. The results of a nationwide population-based cohort study of patients with T2D in Korea (34) showed that sex and diabetes duration exerted interactive effects with DR status in increasing the insomnia risk, and the youngest age group (<40 years) and male patients were most vulnerable to insomnia risk, which is similar to our research findings.

Few studies have focused on the effects of daytime napping on DR, not to mention the combined effects of night sleep and daytime napping on DR. To the best of our knowledge, our study is the first to analyze the separate and combined effects of night sleep and nap on DR among patients with T2D. Patients with both long sleep and long naps exhibited a higher prevalence of DR, which implies superimposed effect on the risk of DR. It is of positive significance to clarify the causal relationship between them and study the potential mechanism, and the findings of our study provide a foundation for future research to build upon. Further studies incorporating longitudinal designs, intervention approaches, detailed metabolic assessments, and a focus on specific patient subgroups will be crucial in advancing our understanding of the complex relationships between sleep and DR in T2D and ultimately translating these findings into improved clinical care and prevention strategies.

The mechanisms between long sleep, long nap, and DR are not fully understood, and the possible mechanisms are as follows: melatonin dysregulation, mainly manifested as low production or mistimed (12, 35); long sleep duration and long nap have been associated with increases in markers of systemic inflammation and adipocytokines, including CRP, IL-6, visfatin, and leptin, which can promote the formation of new blood vessels, increase vascular permeability in the retina, and potentially accelerate the progression of DR by exacerbating retinal ischemia and hemorrhage (36–38); prolonged napping and sleeping can reduce daily energy expenditure, increase appetite, and contribute to obesity (16, 39). Oxidative stress is another key factor linking long sleep and napping to DR. Prolonged sleep and napping may disrupt the balance between reactive oxygen species (ROS) production and antioxidant defenses, leading to oxidative stress. This process could amplify advanced glycation end products (AGEs) accumulation, thickening retinal capillary basement membranes and impairing nutrient diffusion, and exacerbate oxidative damage in the retina (40). Also, these sleep habits might affect retinal microcirculation by altering blood rheology and vascular tone, thereby exacerbating retinal ischemia and hypoxia, and promoting the development of DR (40, 41). In addition, increased insulin resistance, changes in beta-cell function, poor sleep quality, and decreased physical activity are also some of the possible mechanisms (16–18). Given the potential mechanisms mentioned above, exercise, weight loss, increasing anti-inflammatory diets including goji berries, and maintaining a balanced lifestyle overall may be effective treatment options (42–44). In our study, metabolic factors, including SBP, DBP, BMI, and HbA1c, partially explain the mechanism between night sleep, daytime napping, and DR. Previous epidemiological studies have demonstrated that intensive blood glucose and blood pressure control, and improved obesity status could improve the long-term outcomes of patients with T2D, including reducing the risk of DR (45–47). Our study provides novel cross-sectional evidence that prolonged napping and sleeping are positively associated with DR, with observed relationships potentially mediated by blood pressure alterations, obesity indices, and suboptimal glycemic control. These findings highlight the importance of considering sleep patterns in clinical assessments and suggest potential pathways for targeted interventions.

We comprehensively assessed single and overall effects of night sleep and daytime napping on DR. Additionally, our study explored the potential mediating role of metabolic factors on them. However, our study has several limitations. Firstly, duration of sleep and napping is based on patients’ self-report, which may have recall bias and result in potential misclassification. Considering the randomness and non-interference of this issue, we believe that such error would be expected to result toward the null, although this would lead to an underestimation of the risk estimates. Secondly, being limited to a retrospective cross-sectional study, our study failed to explore the longitudinal effects of changes in sleep patterns on DR and further research is needed. Thirdly, our study did not account for potential underlying illness or sleep disturbances (e.g., obstructive sleep apnea and depression) that may influence both sleep patterns and DR. The observed associations between prolonged sleep duration and DR could partially reflect residual confounding from these unmeasured comorbidities and excessive daytime napping might serve as a clinical marker for undiagnosed sleep disorders or systemic inflammation. This potential confounding by indication should be acknowledged when interpreting the findings. Fourthly, it is crucial to recognize that the mediation models assume no unmeasured confounding between the exposure, mediator, and outcome. This assumption is difficult to meet, especially in observational data, and we included mediators in the main model, which may weaken the association. These inherent limitations underscore the need for caution in causal interpretation of mediation effects. In addition, while our study was adjusted for covariates through multivariable regression and performed stratified and conjoint analysis, unmeasured possible confounding variables such as socioeconomic status, genetic predisposition, or dietary habits might introduce residual confounding, which also suggests that future research can further improve the control of these factors.

In summary, long sleep and long nap were associated with DR, and this association was more significant in men and in the diabetes duration < 5 years group. In addition, the effects of night sleep and nap on DR could be superimposed, and that patients with both long sleep and long naps exhibited a higher prevalence of DR. Metabolic factors partially explain the mechanism between night sleep, daytime napping, and DR.

## Data Availability

The original contributions presented in the study are included in the article/**Supplementary Material**. Further inquiries can be directed to the corresponding author.
