# Supplementary material for: Associations of night sleep duration and daytime napping with diabetic retinopathy in patients with type 2 diabetes
Source: Front Endocrinol (Lausanne). 2025 Jun 24;16:1565508. doi: 10.3389/fendo.2025.1565508 (PMC12234332; doi:10.3389/fendo.2025.1565508)
Supplement: Supplementary file 1 [file DataSheet1.docx]

**Supplementary materials**

**Supplementary Table S1.** Associations of extremely short sleep (<6 h/night) with DR events in patients with type 2 diabetes.

| Variables | Model 1 | *P* Value | Model 2 | *P* Value |
| --- | --- | --- | --- | --- |
| Night sleep duration |  |  |  |  |
| Middle sleep | 1.00 (Ref) |  |  |  |
| Extremely short sleep | 1.05 (0.65-1.70) | 0.832 | 0.95 (0.57-1.57) | 0.836 |

Values are expressed as odds ratio (95% confidence interval). Model 1 was adjusted for age and sex; model 2 was further adjusted for smoking, alcohol consumption, SBP, DBP, BMI, HbA1c and duration of diabetes. Middle sleep and no nap were used as references, respectively. *Significant at p < 0.05.


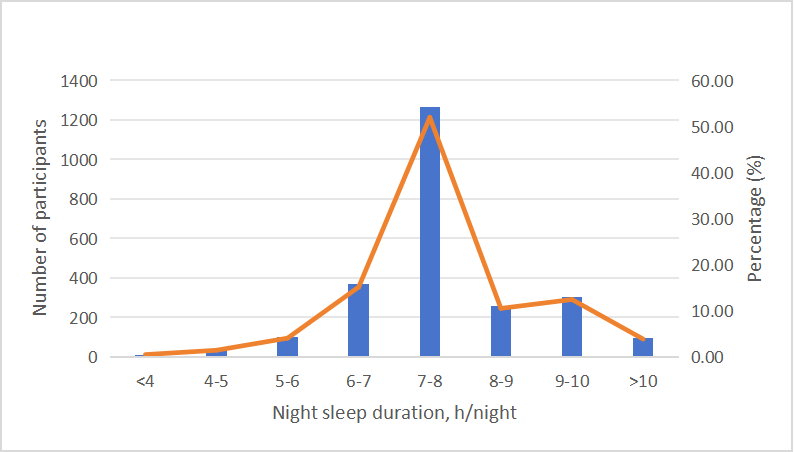


**Supplementary Figure S1. Distribution of study participants stratified by night sleep duration categories.**
